# Extended therapeutic efficacy of dacarbazine following prior treatment with immune checkpoint inhibitors in metastatic melanoma: a single center retrospective study in Korea

**DOI:** 10.12701/jyms.2026.43.14

**Published:** 2026-01-16

**Authors:** Jihyun Na, Jun Young Kim, Seok-Jong Lee, In Hee Lee, Soo Jung Lee

**Affiliations:** 1 Department of Hematology and Oncology, Ulsan University Hospital, University of Ulsan College of Medicine, Ulsan, Korea; 2 Department of Dermatology, Kyungpook National University Hospital, School of Medicine, Kyungpook National University, Daegu, Korea; 3 Lee Seok-Jong’s Dermatology Clinic, Daegu, Korea; 4 Department of Oncology/Hematology, Kyungpook National University Chilgok Hospital, School of Medicine, Kyungpook National University, Daegu, Korea

**Keywords:** Dacarbazine, Immune checkpoint inhibitors, Melanoma, Tumor microenvironment

## Abstract

**Background:**

Immune checkpoint inhibitors (ICIs) have revolutionized the treatment of advanced malignant melanoma. This study examined the real-world efficacy of dacarbazine (dimethyl triazeno imidazole carboxamide, DTIC) in patients who had previously received pembrolizumab, based on the hypothesis that prior ICI treatment enhances the therapeutic response to subsequent chemotherapy.

**Methods:**

This retrospective study included 71 patients with histologically confirmed malignant melanoma treated at Kyungpook National University Chilgok Hospital (Daegu, Korea) between 2011 and 2023. The patients received DTIC for unresectable stage IIIC, IIID, or IV melanoma (American Joint Committee on Cancer, 8th edition).

**Results:**

Among the 71 patients, the median patient age was 64 years (range, 25–89 years). When categorized by melanoma subtype, 18 patients (25.4%) had acral melanoma, 40 (56.3%) had cutaneous melanoma, and 13 (18.3%) had mucosal melanoma. Sixteen of the DTIC-treated patients had not received pembrolizumab previously (DTIC-only group), while 55 had (pem-DTIC group). The median progression-free survival was 2.3 months in the DTIC-only group and 3.9 months in the pem-DTIC group (hazard ratio [HR], 0.246; 95% confidence interval [CI], 0.106–0.576; *p*=0.001). The median overall survival was 6.8 months in the DTIC-only group and 19.0 months in the pem-DTIC group (HR, 0.198; 95% CI, 0.068–0.574; *p*=0.003). The duration of response (DoR) was 4.64 months in the DTIC group and 8.11 months in the pem-DTIC group.

**Conclusion:**

This study demonstrated that the DoR to DTIC was prolonged following ICI therapy (4.64 months vs. 8.11 months). This indicates that prior ICI treatment may enhance tumor sensitivity to chemotherapy, making DTIC a viable option for treating refractory, relapsed, or progressive melanoma.

## Introduction

Melanoma is a highly aggressive skin cancer originating from melanocytes, with an increasing global incidence, particularly in fair-skinned populations. In 2020, melanoma accounted for approximately 325,000 new cases and 57,000 deaths worldwide, according to Global Cancer Statistics [1]. The highest incidence rates have been reported in Australia, New Zealand, North America, and Europe, highlighting the strong correlation between ultraviolet exposure and the risk of melanoma. Although historically more prevalent in Western countries, the incidence of melanoma is increasing in Korea, highlighting the need for greater clinical attention. The advent of immune checkpoint inhibitors (ICIs) has transformed the treatment landscape for advanced malignant melanoma and significantly improved patient outcomes [2]. By modulating immune regulatory pathways, agents such as pembrolizumab enhance the ability of the immune system to effectively target melanoma cells. Pembrolizumab, an anti-programmed cell death protein 1 (PD-1) antibody, has been widely used as a first-line treatment for metastatic melanoma in Korea since 2018, following reimbursement approval based on pivotal findings from the Keynote-006 study [3].

Despite advancements in ICIs, drug resistance remains a major clinical challenge. Approximately 55% of patients with melanoma exhibit innate resistance to single-agent PD-1 inhibitors, while an additional 25% develop resistance within 2 years of treatment initiation [4]. The treatment options for patients with disease progression after pembrolizumab treatment remain limited. Dacarbazine (dimethyl triazeno imidazole carboxamide, DTIC), a chemotherapeutic agent, is one of the few U.S. Food and Drug Administration-approved second-line therapies for metastatic melanoma [5]. Historically, it has demonstrated modest efficacy, with an objective response rate of approximately 20%, a median response duration of 5 to 6 months, and a complete response rate (CR) of 5% [6]. However, DTIC is frequently used as a salvage therapy after anti-PD-1 inhibitor failure, with studies indicating response rates as low as 6% [7] and only transient clinical benefits [8].

Real-world clinical observations suggest that DTIC exhibits greater efficacy when administered after ICI therapy than when administered as a monotherapy. This raises critical questions about prior immune modulation on the effectiveness of subsequent chemotherapy. It has been hypothesized that ICIs prime the tumor microenvironment, increase tumor sensitivity [9], and improve survival outcomes, even in malignancies traditionally resistant to chemotherapy, such as melanoma.

To test this hypothesis, we retrospectively evaluated the real-world efficacy of DTIC in patients with metastatic melanoma who previously received pembrolizumab. Progression-free survival (PFS) and overall survival (OS) were compared between patients treated with DTIC after pembrolizumab therapy and those treated with DTIC alone. Additionally, clinical factors influencing treatment outcomes were analyzed, offering insights into the potential benefits of sequential ICI and chemotherapy strategies for melanoma. This study demonstrated that cytotoxic chemotherapy following ICI treatment prolonged the mean duration of response (DoR) to DTIC.

## Methods

**Ethics statement:** This study was approved by the Institutional Review Board (IRB) of Kyungpook National University Chilgok Hospital (IRB No: KNUCH 2024-01-043). The requirement for informed consent was waived.

### 1. Participants

This retrospective study included 71 patients with histologically confirmed malignant melanoma who underwent chemotherapy between 2011 and 2023 at Kyungpook National University Chilgok Hospital (Daegu, Korea). Patients received either a DTIC or a cisplatin, vinblastine, and DTIC (CVD) regimen for unresectable stage IIIC, IIID, or IV acral, cutaneous, or mucosal melanoma (excluding uveal melanoma), classified according to the American Joint Committee on Cancer 8th edition [9]. The standard DTIC dose was 850 to 1,000 mg/m^2^ every 3 weeks. The CVD regimen comprised 3-week cycles of cisplatin (20 mg/m^2^/day for 4 days), vinblastine (2 mg/m^2^/day for 4 days), and DTIC (800 mg/m^2^ on day 1) [10]. Pembrolizumab was administered at a fixed dose of 200 mg every 3 weeks [11]. The DTIC monotherapy group consisted entirely of patients who received DTIC as a first-line treatment during a period when pembrolizumab was not yet approved in Korea. Data on *c-kit* and *BRAF* status were incomplete. Data were missing for *c-kit* mutations in 53 patients (74.6%) and for *BRAF* mutations in 49 patients (69.0%).

Treatment response was evaluated every 2 or 3 months via chest and abdominopelvic computed tomography. The Response Evaluation Criteria in Solid Tumors version 1.1 (RECIST 1.1) was used to classify responses as CR [12], partial response (PR), stable disease, or progressive disease [13,14].

### 2. Statistical analysis

OS was defined as the time from the initial administration of DTIC to death or the end of the follow-up period (November 15, 2023), whichever occurred first. PFS was measured from the first DTIC administration during radiological disease progression as determined by the RECIST 1.1 criteria. DoR was defined as the time from treatment initiation to disease progression or death in patients who achieved CR or PR.

The overall cumulative survival probability was calculated using the Kaplan-Meier method, and differences in survival rates were assessed using the log-rank test. A two-sided *p*-value of <0.05 was considered statistically significant. Statistical analyses were performed using IBM SPSS ver. 20.0 (IBM Corp., Armonk, NY, USA).

## Results

### 1. Baseline characteristics

Baseline patient characteristics are presented in Table 1. Of the 71 included patients, 26 died by the end of the follow-up period. The median patient age was 64 years (range, 25–89 years). When categorized according to melanoma subtype, 18 patients (25.4%) had acral melanoma, 40 (56.3%) had cutaneous melanoma, and 13 (18.3%) had mucosal melanoma. Sixteen included patients had not received prior pembrolizumab treatment (DTIC-only group). The remaining 55 patients were administered pembrolizumab, followed by DTIC (pem-DTIC group). Baseline characteristics, including age, sex, melanoma subtype, initial disease stage, presence of *BRAF* V600E mutation, *c-kit* mutations, and initial lactate dehydrogenase (LDH) levels [13], were evenly distributed across both groups. Within the cohort, four patients (5.6%) exhibited the *BRAF* V600E mutation, whereas three (4.2%) presented with *c-kit* mutations.

### 2. Progression-free survival and overall survival

Table 2 shows the clinical and treatment parameters associated with PFS and OS. In the univariate analysis of PFS, female sex (hazard ratio [HR], 0.446; 95% confidence interval [CI], 0.246–0.806; *p*=0.008), and the use of pembrolizumab (HR, 0.496; 95% CI, 0.255–0.964; *p*=0.039) were significantly associated with improved outcomes. These factors remained significant in the multivariate analysis, with female sex (HR, 0.299; 95% CI, 0.148–0.602; *p*<0.001) and pembrolizumab treatment (HR, 0.246; 95% CI, 0.106–0.576; *p*=0.001) continuing to demonstrate a positive impact on PFS. In contrast, elevated LDH levels were associated with poorer PFS in univariate analysis (HR, 1.770; 95% CI, 0.946–3.312; *p*=0.074); however, this association was not statistically significant in the multivariate analysis. For OS, the only significant factor identified in the univariate analysis was pembrolizumab use (HR, 0.198; 95% CI, 0.068–0.574; *p*=0.003).

**Fig. 1.**
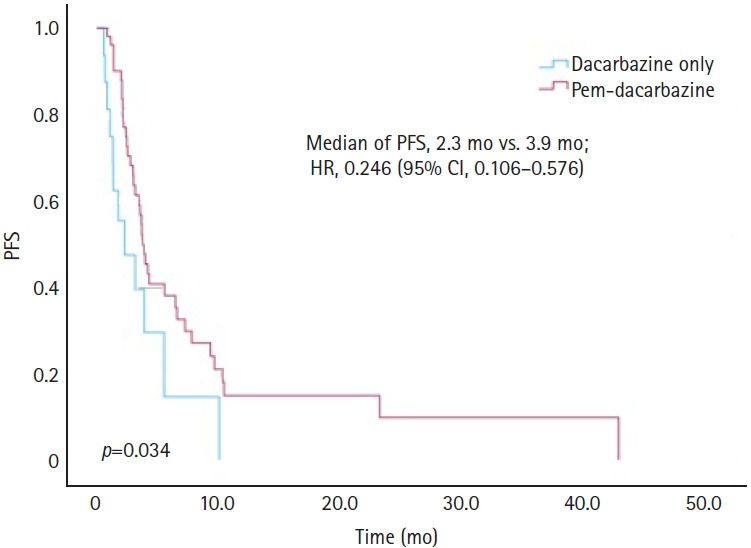
Comparison of progression-free survival (PFS) between dacarbazine monotherapy and dacarbazine treatment following pembrolizumab (Pem) in melanoma. HR, hazard ratio; CI, confidence interval.

**Fig. 2.**
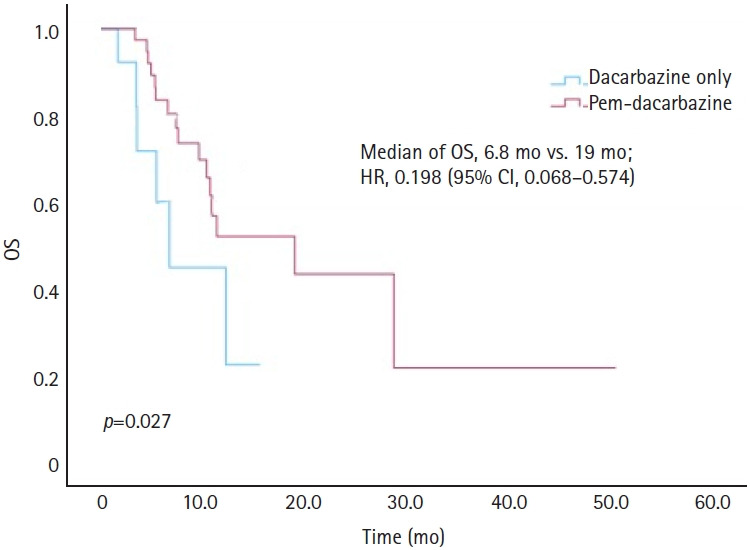
Comparison of overall survival (OS) between dacarbazine monotherapy and dacarbazine treatment following pembrolizumab (Pem) in melanoma.

Fig. 1 illustrates the PFS outcomes using Kaplan-Meier curves for the DTIC-only and pem-DTIC groups. The median PFS was 2.3 months for the DTIC-only group, compared to 3.9 months for the pem-DTIC group, demonstrating a significant difference of 1.6 months (HR, 0.246; 95% CI, 0.106–0.576; *p*=0.034). The OS outcomes are shown in Fig. 2. The median OS was 6.8 months for the DTIC-only group and 19.0 months for the pem-DTIC group, indicating a statistically significant improvement in survival in those who received prior pembrolizumab treatment (HR, 0.198; 95% CI, 0.068–0.574; *p*=0.027).

### 3. Response rate and duration of response

The objective response rate was comparable between the groups, with four patients (25.0%) responding in the DTIC-only group and 14 patients (25.5%) in the pem-DTIC group. However, the disease control rate differed, with five patients (31.2%) achieving disease control in the DTIC-only group compared to 24 patients (43.6%) in the pem-DTIC group (Table 3).

**Fig. 3.**
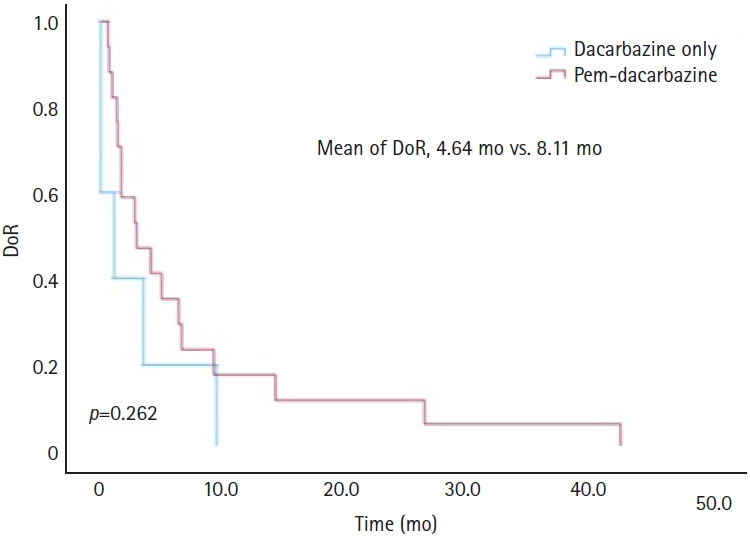
Comparison of duration of response (DoR) between dacarbazine monotherapy and dacarbazine treatment following pembrolizumab (Pem) in melanoma.

The mean DoR for the DTIC-only group was 4.64 months, whereas that for the pem-DTIC group was 8.11 months, indicating that the pem-DTIC group had a longer duration (Fig. 3).

## Discussion

This study highlighted that the DoR for DTIC was prolonged following ICI therapy (4.64 months vs. 8.11 months). In the pem-DTIC group, although the improvement in median PFS was modest (an increase of 1.6 months), the DoR among responders was notably extended by 3.5 months. The prolonged DoR likely contributed to the substantial OS benefit observed, with a difference exceeding 12 months compared with the DTIC-only group.

Although malignant melanoma is considered to be a chemoresistant cancer, there have been reports of exceptional responses to combination chemotherapy with DTIC and cisplatin in patients previously treated with immunotherapy [15]. In our study, two patients who experienced disease progression following pembrolizumab treatment demonstrated notably prolonged PFS after subsequent DTIC therapy. These patients achieved PFS of 25 and 28 months, and both continued to receive DTIC without evidence of disease progression.

The DTIC-induced production of interferon-gamma by natural killer cells triggers major histocompatibility complex class I upregulation, thereby enhancing tumor cell death through cytotoxic T lymphocyte activation [16]. In addition to other cytotoxic effects, DTIC exhibits immunostimulatory properties, especially when combined with other immunotherapeutic agents such as ipilimumab or pembrolizumab [17]. Therefore, DTIC functions as both a cytotoxic agent and an immune modulator. Chemotherapeutic agents, initially selected for their tumoricidal properties, can also influence disease progression through immune-mediated mechanisms, such as promoting lymphocyte infiltration or enhancing extracellular matrix (ECM) remodeling within the tumor microenvironment. Notably, chemotherapy-induced ECM remodeling has been associated with prolonged survival outcomes [18]. A case series demonstrated remarkable responses to conventional chemotherapy after ICI treatment for non-small cell lung cancer [12], which was attributed to modulation of the immunosuppressive cell population within the tumor microenvironment [13]. Specifically, this modulation led to a reduction in the number of regulatory T cells and myeloid-derived suppressor cells, enhancing the antitumor immune response. In a retrospective study on malignant melanoma, Hadash-Bengad et al. [19] reported that patients previously treated with ICIs exhibited a significant improvement in PFS, increasing from 2.5 months in immunotherapy-naïve patients to 5.2 months after ICI treatment. This improvement was associated with increased CD8⁺ T-cell activity, as demonstrated by flow cytometry. This finding suggests that ICIs ‘prime’ the tumor microenvironment, rendering tumors more susceptible to chemotherapy despite ICI treatment failure [20]. Similarly, Bouchereau et al. [8] conducted a retrospective analysis of 72 patients, revealing a PFS of 4.27 months in the ICI-treated group and 2.04 months in the non-ICI group, closely aligning with our findings of 3.9 months and 2.3 months, respectively. In our retrospective study, the overall response rate was 25%, which is consistent with previously reported rates of 20%. The CR rate was 6.3%, which aligns with the established benchmark of 5%. Although DTIC may have served as an immune modulator following ICI therapy, it is plausible that the extended OS and PFS could be attributed, at least in part, to delayed therapeutic effects [21]. Additionally, our findings revealed favorable PFS outcomes for female patients, consistent with existing literature that identifies sex as a significant prognostic factor for survival and relapse in metastatic melanoma [19,20]. The greater benefit observed in female patients may be related to sex-based differences in immune function, including enhanced T-cell-mediated immunity and hormonal modulation, which could augment the immune reactivation induced by DTIC following prior PD-1 blockade [22].

If the overall response rate did not differ significantly, the observed increases in PFS and OS after ICI treatment might be attributed to a subset of patients exhibiting prolonged responses. This extended benefit may be influenced by various factors, including the potential for a sustained response to ICIs. For example, two patients in our cohort maintained PFS for >2 years, with pembrolizumab treatment durations of 9, 12, and 18 months, exceeding the previously reported median PFS of 8.3 months [23]. However, in patients with longer pembrolizumab response durations, subsequent responses to DTIC were comparatively shorter. Given the inherent limitations of small-scale studies, drawing definitive clinical conclusions remains a challenge. Nevertheless, the observed extended efficacy of DTIC after ICI treatment indicates that factors beyond the response duration to pembrolizumab may contribute to these outcomes.

A major limitation of this study is the inclusion of patients treated with the CVD regimen in the DTIC group, which potentially confounded the effects of cisplatin and vinblastine. Additional limitations include the retrospective study design, inherent treatment selection bias, and incomplete data availability. Moreover, the small sample size of patients treated with DTIC alone further restricts the generalizability of our findings. To ensure the robustness and precision of future research, it is essential to conduct larger multicenter studies with more comprehensive patient cohorts. In addition, exploring the potential effects of other conventional chemotherapeutic agents in a post-ICI setting may provide valuable insights. Notably, Goldinger et al. [20] reported that among the various treatment options, the taxane class exhibited the highest efficacy in post-ICI therapy. In the pembrolizumab-paclitaxel plus carboplatin group, only two of 14 patients were evaluated for response, limiting the scope for further analysis. Early treatment discontinuation was common, primarily due to adverse effects such as peripheral polyneuropathy and cytopenia, indicating that this regimen was more toxic than DTIC. Given its favorable tolerability profile, DTIC appears to be a viable treatment option for patients who are older and those with underlying comorbidities, offering a balance between efficacy and manageable toxicity.

In conclusion, DTIC may serve as a viable treatment option for refractory, relapsed, or progressive disease, particularly in patients who have previously exhibited a prolonged response to pembrolizumab. In these patients, DTIC may be an effective chemotherapeutic strategy. Future research should investigate the immunomodulatory effects of DTIC on the tumor microenvironment, which could potentially restore sensitivity to ICI-based therapies.

## Figures and Tables

**Table 1.** The baseline characteristics of patients

Characteristic	DTIC only	Pem-DTIC	*p*-value
No. of patients	16	55	
Age	66.8±10.2	64.7±13.1	0.470
Sex			0.329
Male	11 (68.8)	28 (50.9)	
Female	5 (31.2)	27 (49.1)	
Subtype			0.384
Acral	2 (12.5)	16 (29.1)	
Cutaneous	11 (68.8)	29 (52.7)	
Mucosal	3 (18.8)	10 (18.2)	
*BRAF* V600E mutation			0.052
Yes	0 (0)	4 (9.8)	
No	7 (87.5)	37 (90.2)	
*c-kit* mutation			<0.999
Yes	0 (0)	3 (17.6)	
No	1 (100)	14 (82.4)	
Initial LDH	476.0±309.2	303.2±127.7	0.060
Initial stage			0.144
IIIA	2 (14.3)	2 (4.9)	
IIIB	0 (0)	2 (4.9)	
IIIC	0 (0)	2 (4.9)	
IV	12 (95.7)	41 (91.1)	

Values are presented as number only, mean±standard deviation, or number (%). DTIC, dimethyl triazeno imidazole carboxamide (dacarbazine); Pem, pembrolizumab; LDH, lactate dehydrogenase. DTIC only group: DTIC only without pembrolizumab, Pem-DTIC group: pembrolizumab followed by DTIC. Percentages are calculated based on available data. Missing values are reported in the Methods section.

**Table 2.** Univariate and multivariate analyses of factors associated with progression-free survival and overall survival

Variable	Univariate		Multivariate	
	HR (95% CI)	p-value	HR (95% CI)	p-value
Progression-free survival				
Age (yr) at diagnosis, ≥65 vs. <65	1.614 (0.911–2.819)	0.101		
Female vs. male	0.446 (0.247–0.806)	0.008	0.299 (0.148–0.602)	<0.001
Initial LDH, High vs. Low	1.770 (0.946–3.312)	0.074	1.702 (0.893–3.244)	0.106
*BRAF* mutation, Yes vs. No	1.000 (0.312–3.202)	<0.999		
Subtype, acral vs. mucosal or cutaneous	1.324 (0.710–2.469)	0.378		
Pembrolizumab use Yes vs. No	0.496 (0.255–0.964)	0.039	0.246 (0.106–0.570)	0.001
Overall survival				
Age (yr) at diagnosis, ≥65 vs. <65	1.065 (0.487–2.326)	0.875		
Female vs. male	0.730 (0.331–1.611)	0.436		
Initial LDH, High vs. Low	1.316 (0.567–3.056)	0.522		
*BRAF* mutation, Yes vs. No	0.774 (0.100–6.020)	0.807		
Subtype, acral vs. mucosal or cutaneous	1.779 (0.667–4.747)	0.250		
Pembrolizumab use, Yes vs. No	0.198 (0.068–0.574)	0.003	0.236 (0.079–0.705)	0.010

HR, hazard ratio; CI, confidence interval; LDH, lactate dehydrogenase.

**Table 3.** Response to dacarbazine in both groups

Variable	DTIC only (n=16)	Pem-DTIC (n=55)	*p*-value
Best response			0.669
CR	1 (6.3)	4 (7.3)	
PR	3 (18.8)	10 (18.2)	
SD	1 (6.3)	10 (18.2)	
PD	7 (43.8)	24 (43.6)	
NA	4 (25.0)	7 (12.7)	
Overall response rate (CR+PR)	4 (25.0)	14 (25.5)	0.473
Disease control rate (CR+PR+SD)	5 (31.3)	24 (43.6)	0.434

DTIC, dimethyl triazeno imidazole carboxamide (dacarbazine); Pem, pembrolizumab; CR, complete response; PR, partial response; SD, stable disease; PD, progressive disease; NA, not assessed.
